# Nutritional status and appetite-regulating hormones in early treatment of acute lymphoblastic leukemia among children and adolescents: a cohort study

**DOI:** 10.1590/1516-3180.2019.0307.R1.19112019

**Published:** 2020-06-01

**Authors:** Camila de Carvalho Gomes, Cassia Camila Gomes da Silva, Paulo Ricardo Porfírio do Nascimento, Telma Maria de Araújo Moura Lemos, Aline Marcadenti, Melissa Medeiros Markoski, Ana Paula Trussardi Fayh

**Affiliations:** I MSc. Dietitian, Department of Nutrition, Universidade Federal do Rio Grande do Norte (UFRN), Natal (RN), Brazil.; II BSc. Dietitian, Department of Nutrition, Universidade Federal do Rio Grande do Norte (UFRN), Natal (RN), Brazil.; III PhD. Biologist and Laboratory Technician, Universidade Federal do Rio Grande do Norte (UFRN), Natal (RN), Brazil.; IV PhD. Pharmacist and Assistant Professor, Universidade Federal do Rio Grande do Norte (UFRN), Natal (RN), Brazil.; V PhD. Registered Nutritionist and Professor, Postgraduate Program on Health Sciences (Cardiology), Instituto de Cardiologia, Fundação Universitária de Cardiologia (IC/FUC), Porto Alegre (RS), Brazil; Professor, Postgraduate Program on Nutritional Sciences, Universidade Federal de Ciências da Saúde de Porto Alegre (UFCSPA), Porto Alegre (RS); and Researcher, Instituto de Pesquisa do Hospital do Coração (IP-HCor), São Paulo (SP), Brazil.; VI PhD. Biologist and Professor, Postgraduate Program on Biosciences, Universidade Federal de Ciências da Saúde de Porto Alegre (UFCSPA), Porto Alegre (RS), Brazil.; VII PhD. Dietitian and Associate Professor, Undergraduate Nutrition Program and Stricto Sensu Postgraduate Programs on Physical Education, Nutrition and Health Sciences, Universidade Federal do Rio Grande do Norte (UFRN), Natal (RN), Brazil.

**Keywords:** Body mass index, Diet, Leptin, Ghrelin, Anthropometry, Insulin, Childhood, Food consumption, Cortisol

## Abstract

**BACKGROUND:**

Children with acute lymphoblastic leukemia are at risk of malnutrition, but few studies have described the changes in nutritional status during the different phases of chemotherapy.

**OBJECTIVE:**

To evaluate changes in nutritional status, food intake and appetite-regulating hormones among children and adolescents with acute lymphoblastic leukemia in the first phase of chemotherapy.

**DESIGN AND SETTING:**

Cohort study developed in the pediatric oncology departments of two hospitals in the city of Natal, Rio Grande do Norte, Brazil.

**METHODS:**

Fourteen children/adolescents (mean age of 7 years; 50% female) with acute lymphoblastic leukemia were monitored over the 28 days of an induction chemotherapy cycle. Anthropometric measurements, 24-hours food weight records and appetite-regulating hormone levels (ghrelin, leptin, insulin and cortisol) were obtained at three different times (before, in the middle and at the end of the induction phase).

**RESULTS:**

Most of the patients (85.7%) had normal weight at the beginning of the treatment, and this did not change significantly during the 28 days. Energy and nutrient intakes improved from the start of the treatment to the midpoint, according to the ghrelin levels (from 511.1 ± 8.3 to 519.3 ± 6.6 pg/ml; P = 0.027). Other appetite-regulating hormones did not present changes.

**CONCLUSION:**

Food consumption improves during the first phase of treatment, without alterations in anthropometric nutritional status.

## INTRODUCTION

Pediatric malignancies account for 1% to 3% of cancers diagnosed worldwide, and leukemia is the most common cancer among children, representing about one third of all cancers occurring before the age of 15 years.^1^ Approximately 80% of leukemia cases consist of acute lymphoblastic leukemia,^1^ which is a primary neoplasia of the bone marrow. This is characterized as a heterogeneous group of diseases in which normal medullary and blood elements are replaced by immature cells (blasts) and these cells accumulate in other tissues.^2^ The nutritional status of children with cancer is highly relevant, since good nutritional status enables them to better cope with the intensive cancer treatment regimens.^3^ However, few studies have assessed the nutritional status of these patients during treatment.

Children and adolescents with acute lymphoblastic leukemia experience a spectrum of nutrition-related morbidities during and after treatment.^4^ Weight gain is either a short or a long-term effect of acute lymphoblastic leukemia therapy. The weight gains and body composition changes that are observed during the first four weeks of treatment are associated with administration sf glucocorticoids such as prednisone and dexamethasone.^5^

It has been recognized that survivors in some pediatric cancer groups, including acute lymphoblastic leukemia, present clinical features of metabolic syndrome. These individuals therefore present increased risk factors for cardiovascular disease, such as visceral obesity, insulin resistance, glucose intolerance, dyslipidemia, hypertension and endothelial dysfunction.^6^^,^^7^^,^^8^

Although dietary intake has a direct impact on nutritional status, few studies in homogenous pediatric populations with cancer have assessed this variable.^4^^,^^9^ The absence of a positive correlation between body composition and food consumption has shown the complexity of the energy balance during acute lymphoblastic leukemia. Better understanding of the mechanisms involved in appetite control may lead to development of new therapies, in order to prolong survival in association with better quality of life for these patients.^10^

On the other hand, anorexia and cachexia may also occur in cases of acute lymphoblastic leukemia that are diagnosed during childhood. The possible mediators of anorexia-cachexia syndrome include hormones relating to appetite regulation, such as ghrelin, leptin, cortisol and insulin.^11^ Over the long term, chemotherapeutic agents can also result in changes to leptin secretion, thereby leading to increased plasma levels. However, very few studies have evaluated alterations in ghrelin and leptin during chemotherapy in different types of cancer, and these discrepant results may be due to the different treatments adopted.^12^ Given the risk of malnutrition among children with acute lymphoblastic leukemia,^2^ nutritional assessment at diagnosis and throughout treatment has become an important issue. It has now been pointed out that this is a decisive aspect of successful treatment. Few studies have described the changes in nutritional status that occur during the different phases of chemotherapy, and even fewer have evaluated all the parameters together (body composition, food consumption and biochemical parameters), especially during the first phase.

## OBJECTIVE

The aim of this study was to evaluate nutritional status in relation to appetite-regulating hormones among children and adolescents who had been newly diagnosed with acute lymphoblastic leukemia, before and during the induction treatment phase. The hypothesis was that the first treatment phase would have a negative impact on the nutritional status of these patients.

## METHODS

### Participants and ethics

This was a longitudinal study in which children or adolescents (aged less than 19 years) who had been newly diagnosed with acute lymphoblastic leukemia were included. They had received their diagnoses at the pediatric oncology departments of two hospitals in the city of Natal, Rio Grande do Norte, Brazil, between March and December 2015, and were hospitalized there for the beginning of the induction phase of the chemotherapy treatment.

In total, 17 patients were eligible for inclusion, and no patients or their families refused to participate. After inclusion, three patients left the study because their diagnoses were changed to acute myeloid leukemia. Thus, in the end, 14 patients participated in this study.

Ethical approval was obtained from the ethics committee of the Federal University of Rio Grande do Norte (Universidade Federal do Rio Grande do Norte, UFRN), under protocol no. 976,388, approved on March 7, 2015. All parents or legal guardians signed the written informed consent statement, and children older than six years of age were invited to participate in the study and signed a consent agreement. The study was conducted in accordance with the Declaration of Helsinki.

### Procedures

Chemotherapy for children with acute lymphoblastic leukemia is divided into induction, consolidation, interim maintenance, delayed intensification and maintenance phases.^13^ All the patients in the present study were monitored during the first chemotherapy cycle (induction phase), with nutritional and biochemical measurements that were made at the start (baseline, before chemotherapy), in the middle (after 14 days) and at the end of the induction phase (after 28 days). The goal of induction chemotherapy is to achieve remission. Independent of risk, the children studied here received the following chemotherapy drugs intravenously (IV), intrathecally (IT) and orally: L-asparaginase, methotrexate, prednisone, vincristine and daunorubicin.

### Anthropometric assessment

Weight was measured using a digital scale (Filizola, São Paulo, Brazil) and was recorded to the nearest 0.1 kg. Height was measured using a calibrated stadiometer for infants (Filizola, São Paulo, Brazil), and was recorded to the nearest 0.1 cm. Standard deviation scores for weight, height, weight-for-height and body mass index-for-age were calculated as recommended by the World Health Organization (WHO), to adjust for age and gender.^14^ The cutoff for the diagnosis of underweight was defined as less than the 5^th^ percentile of body mass index-for-age, while a body mass index greater than or equal to the 85^th^ percentile was classified as being overweight. Lastly, a body mass index greater than or equal to the 95^th^ percentile was defined as obesity. Arm circumference and triceps skinfold thickness were measured using a non-stretchable measuring tape (Sanny, São Paulo, Brazil) and a skinfold caliper (Prime Med, São Paulo, Brazil), respectively, and were classified in accordance with the reference standards.^15^ Both measurements were performed in triplicate on the left arm.

### Food consumption

Individual food intake during the hospitalization treatment period was determined by the researchers. All foods and beverages consumed during the day were directly weighed at each of the three evaluation times. Each food or preparation was individually weighed before consumption, using the utensils with which the foods were served and zeroing the scale before adding each food. A portable electronic scale with a capacity of 5 kg and accuracy of 1 g was used for weighing solid foods. Liquid foods were measured in 100 ml and 500 ml graduated cylinders with 1 ml and 10 ml increments, respectively. Any food that was not consumed by each individual was also weighed/measured and was then subtracted from the initial weight.

The food weights were entered into and analyzed using the DietWin Professional software (DietWin Software, Porto Alegre, Brazil) for energy, macronutrients (protein, lipids, cholesterol, carbohydrates and fiber) and micronutrients (vitamin C, vitamin A, vitamin E, vitamin B12, calcium, zinc and iron), primarily taking the Brazilian Table of Food Composition^16^ as the reference.

### Biochemical analyses

Venous samples were collected (10 ml) in the mornings while fasting, at all three different follow-up times. The concentrations of insulin (IU/ml) and cortisol (µg/dl) were analyzed by means of chemiluminescence (active insulin Enzyme-Linked Immunosorbent Assay [ELISA] and assay design cortisol ELISA kits, Labtest, Lagoa Santa, Minas Gerais, Brazil). Ghrelin levels (pg/ml) were analyzed using a total human ghrelin ELISA kit (Sigma-Aldrich, St Louis, Missouri, USA). Leptin levels (ng/ml) were measured using a human leptin ELISA kit (Sigma-Aldrich, St Louis, Missouri, USA). All analyses were carried out in accordance with the manufacturer’s recommended instructions.

### Statistical analyses

The data were analyzed using the Statistical Package for the Social Sciences (SPSS), version 22. A normality test was performed using the Shapiro-Wilk test. Descriptive data were presented as the mean and standard error, for variables with normal distribution. In the case of non-normal distribution, the data were summarized as the median and interquartile range.

The anthropometric data were compared between different times using the chi-square test or Fisher’s exact test. Dietary variables were adjusted for energy intake in accordance with the residual method,^17^ and were analyzed according to the time of data collection using the general estimation equation model. The levels of appetite-regulating hormones were also compared by means of the general estimation equation, with adjustments for age and body weight.

Power estimate analyses regarding the different times (in the middle and at the end of the protocol) were calculated using the WinPepi software, version 11.18. P-values of less than 0.05 were considered statistically significant.

## RESULTS

Fourteen participants were included, among whom six (42.9%) were male. One patient died during the last monitoring period after the intermediate evaluation had been done.

Table 1 shows the anthropometric nutritional assessment results from the patients. The patients generally had adequate anthropometric nutritional status, which was maintained during the follow-up. Height was only measured at the first data collection time, and all the patients had adequate height for their age. At the third evaluation, one patient expressed a desire to not undergo the anthropometric assessment, and this wish was respected. Regarding body mass index/age, it was seen that one patient was underweight at the second data collection time, but had recovered their nutritional status by the time of the third evaluation. The weight-for-age parameter was used for patients under 10 years old, and this did not change during the follow-up. The weight-for-height parameter was used for children under five years of age, and it was noticed that there was a change in one child’s nutritional status (from “at risk of being overweight or obese” to “normal weight”). None of the other anthropometric measurements showed any significant alterations during the follow-up.

**Table 1.** Classification of anthropometric nutritional status at different time during the follow-up on chemotherapy

Variables	Start of treatment (day 0) n (%)	In the middle of treatment (day 14) n (%)	At the end of treatment (day 28) n (%)	P
Height/age				
Adequate height for age	14 (100)	-	-	-
Body mass index/age				
Underweight	-	1 (7.1)	-	0.167
Normal	12 (85.7)	12 (85.7)	11 (91.7)	
Overweight or obesity	2 (14.3)	1 (7.1)	1 (8.3)	
Weight/age^1^				
Adequate weight for age	9 (100)	9 (100)	8 (100)	-
Weight/height^2^				
Underweight for height	-	-	1 (25)	1.000
Normal weight for height	3 (75)	4 (100)	3 (75)	
At risk of being overweight or obese	1 (25)	-	-	
Arm circumference (cm)^3^				
Risk of deficit or low fat storage	4 (36.4)	4 (35.4)	4 (40)	0.083
Adequate	7 (63.6)	7 (63.6)	6 (60)	
Arm muscle circumference (cm)^3^				
Risk of deficit or low fat storage	6 (54.5)	6 (60)	5 (55.6)	1.000
Adequate	5 (45.5)	4 (40)	4 (44.4)	
Triceps skinfold (mm)				
Adequate	8 (57.1)	7 (53.8)	5 (45.5)	0.080
Risk of deficit or low fat storage	6 (42.9)	6 (46.2)	6 (54.5)	

^1^For children 0 to < 10 years old; ^2^for children 0 to < 5 years old; ^3^n = 11 because some assessments were not performed (children with venous access or refusal).

P-value with Fisher’s exact test before and at the end of the induction phase.

**Figure 1.**
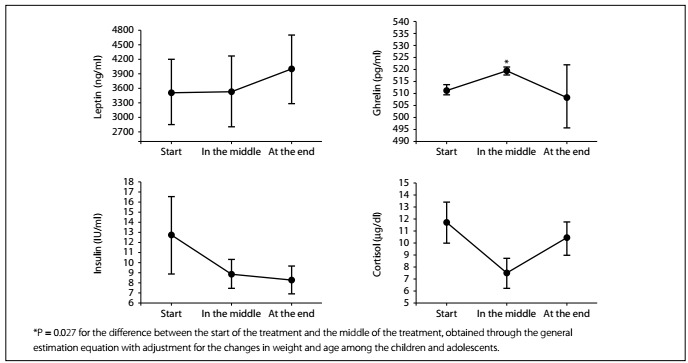
Concentrations of appetite-regulating hormones at the different treatment times: start of treatment (day 0), in the middle of treatment (day 14) and at the end of treatment (day 28).

Table 2 shows the results relating to food consumption during the chemotherapy induction phase. Overall, there were increases in the consumption of all macronutrients, cholesterol, fiber, vitamin B12 and iron, and in energy intake This enhancement of food consumption was in line with the increase in plasma ghrelin concentration between the baseline and the second evaluation (P = 0.027; power analysis 99.3%). At the last evaluation, there was a non-significant difference with a low power analysis value (41.2%) (Figure 1). Although a reduction in cortisol concentration had been expected, its levels did not show any significant reduction from the beginning to the end of the induction cycle. Likewise, insulin concentration hardly changed between the evaluations. A similar response was seen in relation to serum leptin, which did not show any significant changes during the treatment.

**Table 2.** Food intake of patients at different times during the follow-up on chemotherapy

Variables	Start of treatment (day 0) M ± SE	At the middle of treatment (day 14) M ± SE	At the end of treatment (day 28) M ± SE	P
Energy intake (kcal)	1330.7 ± 176.1^a^	1845.2 ± 191.7^b^	1692.9 ± 244.6^a,b^	0.01
Carbohydrate intake (g)	208.3 ± 7.6^a^	293.9 ± 5.7^b^	214.7 ± 10.4^a,c^	< 0.001
Protein intake (g)	34.7 ± 2.2^a^	58.9 ± 3.0^b,d^	59.9 ± 5.2^c,d^	< 0.001
Lipid intake (g)	24.2 ± 2.1^a^	39.5 ± 2.2^b,d^	45.1 ± 6.8^c,d^	< 0.001
Cholesterol intake (mg)	141.0 ± 25.0^a,b^	273.4 ± 41.7^b,c^	182.4 ± 25.9^a,c^	0.02
Fiber intake (g)	14.8 ± 1.7^a^	19.3 ± 2.11^a,b^	12.0 ± 2.0^a^	0.01
Vitamin A intake (mcg)	1076.9 ± 251.5	1607.7 ± 369.2	1118.9 ± 511.0	0.41
Vitamin C intake (mg)	256.7 ± 147.8	256.7 ± 147.8	203.9 ± 64.9	0.76
Vitamin E intake (mg)	4.4 ± 0.7	4.4 ± 0.8	5.9 ± 1.1	0.45
Vitamin B12 intake (mcg)	0.9 ± 0.2^a^	1.8 ± 0.3^b^	1.8 ± 0.3^b^	0.01
Zinc intake (mg)	5.2 ± 2.2	5.1 ± 0.5	5.2 ± 0.8	0.99
Iron intake (mg)	5.9 ± 0.5^a^	8.9 ± 0.8^b^	7.3 ± 0.7^b^	0.03
Calcium intake (mg)	414.8 ± 66.9	510.4 ± 54.0	604.2 ± 68.3	0.05

M = mean; SE = standard error.

P-value obtained through general estimation equation; ^a,b,c,d^Different letters means differences between times according to energy/nutrient intake.

## DISCUSSION

The importance of early identification of nutritional risk and appropriate management of nutrition among hospitalized children at admission is well understood. Numerous screening tools have been developed for this population.

Some cancer patients experience weight loss before diagnosis and during their treatment.^6^^,^^7^ This problem particularly affects patients with solid tumors and medulloblastoma.^8^ However, the majority of the patients in the present study did not present any changes in their anthropometric nutritional status parameters that had been measured at the time of the diagnosis, given that there were no significant changes in these parameters over the 28 days of the chemotherapy induction phase.

Our findings are consistent with those of other studies in the literature.^7^^,^^8^^,^^11^ Those studies showed that the frequency of malnutrition in relation to the hematological system was low at the time of the cancer diagnosis, probably due to the acute nature of the disease. More recently, even with the presence of a catabolic disease like acute lymphoblastic leukemia, many studies have shown increased frequency of overweight and obesity among newly diagnosed patients. Ladas et al.^18^ evaluated the nutritional status and food intake of 640 children with acute lymphoblastic leukemia and found that 27% were overweight or obese at the time of the diagnosis. In the study by Tan et al.,^19^ the frequency of overweight was 24.5% among 53 children with acute lymphoblastic leukemia and acute myeloid leukemia. In our study, we found a similar frequency (21.4%), which was also in accordance with other studies on patients with hematological cancer.^6^^,^^7^ It is now known that the presence of obesity affects the prognosis, such that it increases mortality among children and adolescents with acute lymphoblastic leukemia.^20^

The anthropometric nutritional status of our patients was preserved during the induction period, thus differing from previous studies in the literature. According to Brinksma et al.,^8^ this weight loss prior to diagnosis may have been due to reduced energy intake, increased energy requirements or altered metabolism. Lindemulder et al.^11^ followed up 269 children and adolescents from the time of diagnosis until the maintenance phase of treatment and found that there was a significant increase in body mass index between the time of the diagnosis and the consolidation phase of cancer treatment, but especially during the first month of treatment. In following up patients with hematological cancer, Zareifar et al.^9^ observed late changes in nutritional status, especially six months after treatment had started. These differences may have been due to the sample sizes in these studies and the different chemotherapy protocols used, with different doses of corticosteroids. They may also have been the result from increased energy intake combined with reduced physical activity.^21^

Epidemiological studies have frequently used body mass index to define obesity and explore its association with cancer risk and mortality.^20^ However, associations with other anthropometric markers are also important. Anthropometric measurements of fat reserves may be more sensitive to changes in nutritional status than body mass index.^6^^,^^19^^,^^22^^,^^23^

In the present study, it was found that most patients had an adequate body mass index in all the treatment phases, but half of them had low fat reserves, as measured using the triceps skinfold. Similar findings, with high frequency of body fat depletion ascertained through anthropometric techniques, were reported by Dávila et al.^6^ and Ani.^7^

Even without significant alterations during the evaluation period, these indicators should not be underestimated, since they contribute towards assessing patients’ body composition in the absence of more sophisticated techniques.^24^ The short follow-up time of the present study (28 days) was possibly not enough to reverse the changes in fat stores and muscle mass that were measured through anthropometric techniques.

Regarding the food consumption assessment, in which all food and drink consumed during three 24-hour evaluations was weighed and measured, this form of assessment was novel, to the best of our knowledge. It reduced the measurement errors that are usually seen in studies evaluating food intake and increased the accuracy of energy intake measurements, in addition to minimizing underreporting. However, it was difficult to compare the results from our study with other results because the data available have usually come from a 24-hour recall or a dietary diary, which are less accurate and more questionable. Moreover, it was difficult to draw uniform conclusions about the adequacy of dietary intake among childhood cancer patients because intake has been assessed at different time points and because different norms have been used.^25^

Energy intake in some studies has been assessed in relation to recommended daily allowances, whereas in other studies it has been compared with the energy intake of healthy controls. Only one cross-sectional study assessed energy intake against calculated individual requirements,^26^ as done in the present study. In that study, the consumption of nutrients among children with hematological malignancies was similar to what was seen in the present study.

We did not use food adequacy data, given the large variation in the ages of the samples in previous studies. Some studies have used 80% of the recommended daily allowances as a cutoff value for determining intake adequacy,^25^ but a better strategy would probably consist of comparing the intake of childhood cancer patients with that of healthy controls. This may also constitute a limitation of the present study, because the food intake that was measured was not consumed in the children’s own homes or in places where they were normally fed. Therefore, this does not represent their usual consumption, considering that these children might have had a dietary intake that differed from what was normal for them, given that they were being carefully watched. However, the consumption measurements were always made at the same place in the three evaluations, and the amounts and types of foods were the same, which will have improved the comparison between the evaluation times.

An increase in energy and macronutrient intake during the induction phase was observed in the present study, possibly because the children were stressed or lacking in appetite because of the time that elapsed before the chemotherapy started. This increase was also mentioned in another study that evaluated patients with cancer over a one-year period,^25^ and also in an evaluation on the intake among children with acute lymphoblastic leukemia during the induction phase, compared with a healthy control group.^21^

In contrast with the amount of data in the literature regarding energy intake, there is a scarcity of data in the literature regarding protein requirements during childhood. Proteins are essential for growth and synthesis of lean body mass.^22^ Protein requirements are assumed to increase during illness, so as to compensate for muscle deterioration, which is caused by inflammation and inactivity.^27^ Some studies (like the present study) have shown that protein intake among patients exceeds the daily recommendations after chemotherapy is started.^25^^,^^28^ One possible reason for this increase in energy and protein intake after treatment is started might be the use of anabolic steroids, particularly glucocorticoids, which are characteristically administered to patients with acute lymphoblastic leukemia, given its anabolic nature.^29^

Ghrelin, leptin and insulin are hormones relating to food intake regulation and consequently to body weight control.^30^ Cortisol mobilizes energy, increases cerebral perfusion and glucose utilization and enhances cardiovascular function to help individuals adjust to real or perceived threats.^31^ In the present study, we evaluated these hormones in addition to the eating behavior response among patients with acute lymphoblastic leukemia. Aside from the ghrelin levels (which significantly increased at the second assessment), no other statistical difference was observed. Changes to the levels of these hormones are associated with chronic exposure to diets with low caloric content,^22^ which was not the case in the present study, in which patients were followed up for a relatively short period of time and showed adequate energy intake. For example, in the study by Adam et al.,^32^ the fasting insulin levels significantly increased during the first year of chemotherapy. Another possible reason why no changes to the levels of these hormones were seen in the present study may have been the absence of energy restrictions among our population over this period.^33^

According to Mariani et al.,^34^ use of chemotherapeutic agents can also result in modified leptin secretion over the long term, thus leading to increased plasma levels. However, the behavior of leptin reported in studies available in the literature has varied considerably. In the study by Park et al.,^35^ children with pediatric cancer showed higher plasma leptin concentrations than those of healthy children, but lower plasma ghrelin levels. Acute leukemia-related inflammation and serum hyperlipidemia may suppress ghrelin at the time of diagnosing childhood acute lymphoblastic leukemia if the inflammatory indices are abnormal, i.e. when low-grade inflammation is present. A recent study from our group showed that children with acute lymphoblastic leukemia presented reduced inflammation and oxidative stress during the induction period.^33^

Moschovi et al.^36^ followed up nine pediatric acute lymphoblastic leukemia patients from diagnosis to the maintenance phase, and no significant decrease in leptin levels was observed in these patients. These discrepant results may have been due to the body weight changes and food consumption of these patients. The increased ghrelin levels observed in the present study , although small, coincide with the results from the study by Moschovi et al.,^3^ in which nine patients with leukemia were evaluated and a notable increase in these hormone levels was observed after the eighth chemotherapy cycle. Increased ghrelin levels coincided with the increased food intake of the patients in the present study, and it can be suggested that a cause-effect mechanism may exist. However, few studies have evaluated ghrelin alterations during chemotherapy among acute lymphoblastic leukemia pediatric patients to help support this hypothesis. The small sample size of our study does not allow us to generalize the data, especially because of the low statistical power.

The strong point of the present study is that it included many variables relating to nutritional status (body composition, food consumption and biochemical parameters). Moreover, this study made it possible to follow up patients over a 24-hour period at three different times during the induction phase. Thus, food consumption could be evaluated through weighted recordings, and potential confounders were controlled for.

On the other hand, the types of patient evaluated in the present study are rare, even in large oncological institutions. Therefore, it is obvious that the patients in our study may have represented heterogeneous groups, and the small sample size reflects this. Hence, this is another limitation of our study: the small sample size had the consequence that it was unclear whether ethnic/racial diversity or cultural factors may have played a role in eating behavior.

Nonetheless, our study is one of the few prospective cohort studies describing the changes in nutritional status among pediatric patients with acute lymphoblastic leukemia. Although the sample size was small, the longitudinal design of the study, its low dropout rate and its results are useful for developing nutritional strategies to improve the outcomes among children with cancer.

## CONCLUSION

Increases in food consumption and ghrelin concentration were observed during the induction period for treating acute lymphoblastic leukemia, but without recovering the patients’ anthropometric status. Other appetite-regulating hormones did not undergo changes. Our initial hypothesis that the first treatment phase could have a negative impact on the patients’ nutritional status was rejected. The relative improvement in dietary consumption may have been related to the hormonal response or to the pharmacological therapy for acute lymphoblastic leukemia. Further studies with a similar design but larger sample size should be conducted to confirm these results among acute lymphoblastic leukemia patients.
